# Efficacy of Front-Line Ibrutinib and Rituximab Combination and the Impact of Treatment Discontinuation in Unfit Patients with Chronic Lymphocytic Leukemia: Results of the Gimema LLC1114 Study

**DOI:** 10.3390/cancers14010207

**Published:** 2021-12-31

**Authors:** Francesca Romana Mauro, Francesca Paoloni, Stefano Molica, Gianluigi Reda, Livio Trentin, Paolo Sportoletti, Monia Marchetti, Daniela Pietrasanta, Roberto Marasca, Gianluca Gaidano, Marta Coscia, Caterina Stelitano, Donato Mannina, Nicola Di Renzo, Fiorella Ilariucci, Anna Marina Liberati, Lorella Orsucci, Francesca Re, Monica Tani, Gerardo Musuraca, Daniela Gottardi, Pier Luigi Zinzani, Alessandro Gozzetti, Annalia Molinari, Massimo Gentile, Annalisa Chiarenza, Luca Laurenti, Marzia Varettoni, Adalberto Ibatici, Roberta Murru, Valeria Ruocco, Ilaria Del Giudice, Maria Stefania De Propris, Irene Della Starza, Sara Raponi, Mauro Nanni, Paola Fazi, Antonino Neri, Anna Guarini, Gian Matteo Rigolin, Alfonso Piciocchi, Antonio Cuneo, Robin Foà

**Affiliations:** 1 Hematology, Department of Translational and Precision Medicine, Sapienza University, Via Benevento 6, 00161 Rome, Italy; ruocco.1584726@studenti.uniroma1.it (V.R.); delgiudice@bce.uniroma1.it (I.D.G.); depropris@bce.uniroma1.it (M.S.D.P.); dellastarza@bce.uniroma1.it (I.D.S.); raponi@bce.uniroma1.it (S.R.); nanni@bce.uniroma1.it (M.N.); guarini@bce.uniroma1.it (A.G.); rfoa@bce.uniroma1.it (R.F.); 2 GIMEMA Foundation, 00187 Rome, Italy; f.paoloni@gimema.it (F.P.); p.fazi@gimema.it (P.F.); a.piciocchi@gimema.it (A.P.); 3 Department of Hematology, Pugliese Ciaccio Hospital, 88100 Catanzaro, Italy; smolica@libero.it; 4 Hematology Department, Foundation IRCCS Ca’ Granda, Ospedale Maggiore Policlinico, University of Milan, 20100 Milan, Italy; gianluigi.reda@policlinico.mi.it (G.R.); antonino.neri@unimi.it (A.N.); 5 Hematology Division, Department of Medicine, University of Padua, 35128 Padua, Italy; livio.trentin@unipd.it; 6 Institute of Hematology-Centro di Ricerca Emato-Oncologica (CREO), Department of Medicine, University of Perugia, 06129 Perugia, Italy; paolo.sportoletti@unipg.it; 7 Department of Hematology, SS. Antonio e Biagio e Cesare Arrigo Hospital and University of Eastern Piedmont, 15121 Alessandria, Italy; moniamarchettitamellini@gmail.com; 8 Department of Hematology, Azienda Ospedaliera SS Arrigo e Biagio e Cesare Arrigo, 15121 Alessandria, Italy; dpietrasanta@ospedale.al.it; 9 Hematology Unit, Department of Medical and Surgical Sciences, University of Modena and Reggio Emilia, 41126 Modena, Italy; roberto.marasca@unimore.it; 10 Division of Hematology, Department of Translational Medicine, University of Eastern Piedmont, AOU Maggiore della Carità, 28100 Novara, Italy; gianluca.gaidano@med.uniupo.it; 11 Division of Hematology, A.O.U. Città della Salute e della Scienza di Torino and Department of Molecular Biotechnology and Health Sciences, University of Torino, 10100 Torino, Italy; marta.coscia@unito.it; 12 Department of Hematology, Azienda Ospedaliera Bianchi Melacrino Morelli, 89124 Reggio Calabria, Italy; caterinastelitano27@gmail.com; 13 Division of Hematology, Azienda Ospedaliera Papardo, 98158 Messina, Italy; donamanni@gmail.com; 14 Hematology and Stem Cell Transplant Unit, “Vito Fazzi” Hospital, 73100 Lecce, Italy; direnzo.ematolecce@gmail.com; 15 Hematology, Azienda Ospedaliera Arcispedale Santa Maria Nuova IRCCS, 42123 Reggio Emilia, Italy; Fiorella.Ilariucci@ausl.re.it; 16 Department of Hematology, Università degli Studi di Perugia, A.O.S., 05100 Terni, Italy; marina.liberati@unipg.it; 17 Department of Hematology, Azienda Ospedaliera Universitaria Città della Salute e della Scienza di Torino, 10100 Torino, Italy; lorsucci@cittadellasalute.to.it; 18 Hematology and Bone Marrow Transplant Center, Azienda Ospedaliera Universitaria di Parma, 43126 Parma, Italy; fRe@ao.pr.it; 19 Division of Hematology, Santa Maria delle Croci Hospital, 48121 Ravenna, Italy; monica.tani@auslromagna.it; 20 Istituto Scientifico Romagnoli per lo Studio e la Cura dei Tumori-IRST, 47014 Meldola, Italy; gerardo.musuraca@irst.emr.it; 21 A.O.U. S. Giovanni Battista A.O. Mauriziano-Umberto I, 10128 Torino, Italy; dgottardi@mauriziano.it; 22 IRCCS Azienda Ospedaliero-Universitaria di Bologna-Istituto di Ematologia “Seràgnoli” and Dipartimento di Medicina Specialistica, Diagnostica e Sperimentale, Università di Bologna, 40138 Bologna, Italy; pierluigi.zinzani@unibo.it; 23 Hematology, Department of Medicine, Surgery and Neurosciences, University of Siena, 53100 Siena, Italy; gozzetti@unisi.it; 24 Department of Oncology and Hematology, Infermi Hospital, 47923 Rimini, Italy; annalia.molinari@auslromagna.it; 25 Hematology Section, Cosenza Hospital, 87100 Cosenza, Italy; massim.gentile@tiscali.it; 26 Division of Haematology, Azienda Policlinico-OVE, 95123 Catania, Italy; annalisa.chiarenza@gmail.com; 27 Fondazione Policlinico Universitario A Gemelli, IRCCS, 00168 Rome, Italy; Luca.Laurenti@unicatt.it; 28 Division of Hematology, Fondazione IRCCS Policlinico San Matteo, 27100 Pavia, Italy; m.varettoni@smatteo.pv.it; 29 U.O. Ematologia e Centro Trapianti di Midollo, Policlinico San Martino, 16132 Genova, Italy; adalberto.ibatici@hsanmartino.it; 30 Hematology and Stem Cell Transplantation Unit, Ospedale A. Businco, ARNAS “G. Brotzu”, 34121 Cagliari, Italy; roberta.murru@tiscali.it; 31 Hematology Section, St. Anna University Hospital, 44121 Ferrara, Italy; rglgmt@unife.it (G.M.R.); cut@unife.it (A.C.)

**Keywords:** chronic lymphocytic leukemia, treatment, ibrutinib, rituximab, unfit, adverse events

## Abstract

**Simple Summary:**

This prospective, multicenter study aimed to investigate the efficacy and safety of a front-line treatment with the ibrutinib and rituximab combination in 146 unfit patients with chronic lymphocytic leukemia (CLL). We observed an OR, CR, and 48-month PFS rates of 87%, 22.6%, and 77%, respectively. Responses with undetectable MRD were observed in 6.2% of all patients and 27% of CR patients. TP53 disruption and B-symptoms revealed a significant and independent impact on PFS. The 48-month cumulative treatment discontinuation rate due to adverse events in this patient population was 29.1%. It was significantly higher in male patients, in patients aged ≥70 years, and in those managed at centers that enrolled less than five patients. In conclusion, the ibrutinib and rituximab combination was an effective front-line treatment for unfit patients with CLL. However, a high rate of treatment discontinuations due to adverse events was observed in this unfit population.

**Abstract:**

The GIMEMA group investigated the efficacy, safety, and rates of discontinuations of the ibrutinib and rituximab regimen in previously untreated and unfit patients with chronic lymphocytic leukemia (CLL). Treatment consisted of ibrutinib, 420 mg daily, and until disease progression, and rituximab (375 mg/sqm, given weekly on week 1–4 of month 1 and day 1 of months 2–6). This study included 146 patients with a median age of 73 years, with IGHV unmutated in 56.9% and *TP*53 disrupted in 22.2%. The OR, CR, and 48-month PFS rates were 87%, 22.6%, and 77%, respectively. Responses with undetectable MRD were observed in 6.2% of all patients and 27% of CR patients. *TP*53 disruption (HR 2.47; *p* = 0.03) and B-symptoms (HR 2.91; *p* = 0.02) showed a significant and independent impact on PFS. The 48-month cumulative rates of treatment discontinuations due to disease progression (DP) or adverse events (AEs) were 5.6% and 29.1%, respectively. AEs leading more frequently to treatment discontinuation were atrial fibrillation in 8% of patients, infections in 8%, and non-skin cancers in 6%. Discontinuation rates due to AEs were higher in male patients (HR: 0.46; *p* = 0.05), patients aged ≥70 years (HR 5.43, *p* = 0.0017), and were managed at centers that enrolled <5 patients (HR 5.1, *p* = 0.04). Patients who discontinued ibrutinib due to an AE showed a 24-month next treatment-free survival rate of 63%. In conclusion, ibrutinib and rituximab combination was an effective front-line treatment with sustained disease control in more than half of unfit patients with CLL. Careful monitoring is recommended to prevent and manage AEs in this patient population.

## 1. Introduction

Chronic lymphocytic leukemia (CLL) is the most common leukemia in the adult population. About 21,250 new cases of CLL have been estimated in the United States for 2021. CLL mainly affects aged subjects, with an average age at diagnosis of around 70 years [1]. During the last years, relevant advances in the understanding of the biologic mechanisms associated with the proliferation and survival of CLL cells have led to the clinical use of ibrutinib, a small molecule that inhibits the Bruton tyrosine kinase (BTK). From the first studies, ibrutinib has been proven to be highly effective, regardless of age, prior treatment, and high-risk biologic features of the leukemic cell [2,3]. After that, several randomized trials demonstrated the superiority over chemoimmunotherapy of front-line ibrutinib as a single agent, or combined with an anti-CD20 monoclonal antibody [4,5,6,7]. The excellent therapeutic activity of this agent has revolutionized the treatment approach of CLL, and today, ibrutinib is a standard of care for CLL patients of all ages, both in the relapsed/refractory and in front-line settings. However, despite the excellent response rates and prolonged responses, treatment discontinuation, mainly due to adverse events (AE), is a relevant problem limiting the effectiveness of this agent [8,9,10].

Based on the improved outcomes observed with the addition of rituximab to chemotherapy [11,12,13,14] and on the efficacy of ibrutinib and rituximab [15], the GIMEMA (Gruppo Italiano Malattie EMatologiche dell’Adulto) group, in 2015, started a prospective, multicenter study to investigate the safety and efficacy of a front-line treatment, consisting of six courses of the ibrutinib and rituximab combination followed by ibrutinib single agent, in unfit patients with CLL. Herein, we report the long-term results of this schedule in 146 unfit patients with CLL, the safety profile of treatment, and the reasons and prognostic impact of treatment discontinuation.

## 2. Methods

### 2.1. Patients

Between March 2015 and April 2017, 159 unfit patients with CLL were enrolled in the GIMEMA LLC1114 study, a prospective, phase 2, multicenter, single-arm study. Inclusion criteria included previously untreated CLL requiring treatment according to the International Workshop on CLL (iwCLL) criteria [16]. Patients were defined as unfit in the presence of a Cumulative Illness Rating Scale (CIRS) [17] score >6, and or a creatinine clearance <70 mL/min.

In addition, the absence of Richter transformation, active infection, or secondary malignancy was also required in patients enrolled in the study. The assessment of the biologic profile included fluorescence-in-situ-hybridization (FISH) and the IGHV and *TP53* mutation status as previously described [18,19].

### 2.2. Treatment

Treatment consisted of ibrutinib, 420 mg once daily given continuously, and rituximab, 375 mg/sqm, every week, on day 1 of month 1 and day 1 of months 2–6. Patients received ibrutinib single agent until one of the following events disease progression or severe toxicity, or for a maximum of 6 years.

All patients received *Pneumocystis carinii* prophylaxis with trimethoprim-sulfamethoxazole.

### 2.3. Response

The response was assessed according to the iwCLL criteria [16] 2 months after the last administration of rituximab. The response assessment included clinical examination, PB examination, BM aspirate and biopsy, and total body CT scan. In patients who achieved a complete response (CR), a centralized assessment of MRD, in both the PB and BM, was performed by an eight-color flow cytometry assay with a sensitivity of at least 10^–4^ according to the internationally standardized European Research Initiative on CLL criteria [20]. MRD was further assessed in PB and BM by allele-specific oligonucleotide polymerase chain reaction (PCR) in patients in complete remission (CR) with undetectable MRD (uMRD) by flow cytometry. The response was monitored every 6 months during the follow-up.

### 2.4. Study Endpoints

The primary endpoint of the study was progression-free survival (PFS). The secondary endpoints included the overall responses rate (ORR), the CR rate, the rate of CRs with undetectable MRD (uMRD) in the PB and BM, overall survival (OS), and survival outcomes according to the clinical and biological features of the patients. The safety profile and reasons for permanent discontinuation of treatment were also analyzed. AEs were graded according to the Common Terminology Criteria for Adverse Events, version 3 [21].

### 2.5. Statistical Analysis

Patients’ characteristics were summarized using cross-tabulations for categorical variables or using quantiles for continuous variables. In the univariate analysis, non-parametric tests were performed for comparisons between groups (Chi-Squared and Fisher Exact test in the case of categorical variables or response rate; Mann−Whitney and Kruskal−Wallis tests in case of continuous variables). Survival distributions were estimated using the Kaplan−Meier Product Limit estimator. Differences in survival curves were evaluated using the Log-Rank test. Cox regression models were performed in univariate and multivariate analyses to assess the effect of clinical and biologic factors on PFS and OS. Hazard ratios (HR) and a 95% confidence interval were reported as parameter results of the Cox regression models. The multivariate models were all considered relevant.

Curves of the cumulative incidence of treatment discontinuations by specific causes (e.g., adverse events) were estimated using the proper non-parametric method. The Gray test was applied for comparing the curves of cumulative incidence and the Fine and Gray regression model was used in the univariate and multivariate analyses to assess the effects of covariates on the survival outcome in cases of competitive risks.

All analyses were analyzed on an Intention-To-Treat basis. All tests were two-sided, accepting *p* < 0.05 as indicating a statistically significant difference. Confidence intervals were calculated at the 95% level. All of the analyses were performed using the SAS software (release 9.4; SAS Campus Drive, Cary, NC 27513, USA) and R system software (R Foundation for Statistical Computing c/o Institute for Statistics and Mathematics, Wirtschaftsuniversität, 1020 Wien, Austria). Details about data collection are reported in Supplementary Figure S1.

### 2.6. Ethics

This study has been carried out according to the Helsinki Declaration and was approved by the Ethical Committees of all of the participating institutions. All of the participants gave their written informed consent. This study is registered at ClinicalTrials gov, Identifier: NCT02232386.

## 3. Results

One hundred and fifty-nine CLL patients were enrolled in this study. The patient disposition is described in Supplementary Figure S1. Thirteen patients were considered not eligible and were excluded from the study before receiving any study drug (not eligible, 8; AE, 2; death, 1; refused treatment, 1; medical decision, 1). One hundred and forty-six patients with a median follow-up of 49.1 months (IQR, 39.4–54) represent the intention-to-treat population assessed for treatment response and safety. The baseline clinical and biological characteristics of patients are summarized in Table 1. Briefly, the median age was 73 years (range 37–88), the median CIRS score 6, the median creatinine clearance 62.7 mL/min, and 37.9% of the patients had an ECOG performance score of 1–2. Unmutated IGHV status was observed in 56.9% of patients and *TP53* disruption (del17p and/or *TP53* mutation) in 22.2%.

### 3.1. Response to Treatment

Patients received a median number of six courses of the ibrutinib and rituximab combination (range 1–6), and 137 (94%) completed the planned six courses of treatment.

Here, 127/146 patients (87%) achieved a response at the end of the ibrutinib and rituximab combination. Responses were confirmed by CT scan and included a complete response (CR/CRi) in 33 (22.6%) patients, a partial response (PR) in 76 (52.1%), and a PR with lymphocytosis (PR-L) in 18 (12.3%).

In an ITT analysis, 9 of the 146 patients (6.2%) obtained a flow-cytometric uMRD at one or more time points, three at the EOCT, and six during the follow-up. When the analysis was restricted to the 33 patients with CR, the rate of patients with uMRD was 27.3% (9/33). While uMRD was transient in five patients, in four (4/146, 2.7%; 4/33 patients with CR, 12.1%) it persisted for 6, 52, 52, and 54 months. Three of the nine patients with uMRD by flow-cytometry showed no residual disease in the PB also by ASO-PCR at one or more time points.

Ten (6.9%) patients showed stable disease, one progressed (0.6%) while eight (5.5%) discontinued the ibrutinib and rituximab combination (adverse event, 7; second malignancy, 1).

### 3.2. Survival Analysis

**Figure 1:**
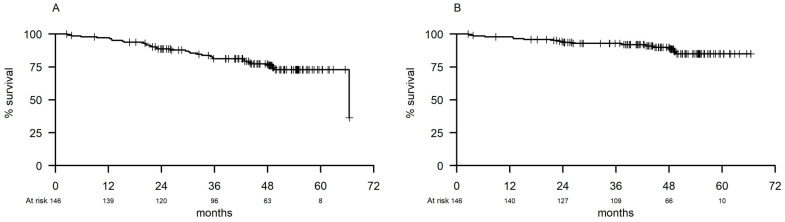
(**A**) Progression-free survival. (**B**) Progression-free survival by *TP*53 disruption.

Ten (6.9%) patients developed disease progression (CLL progression, 9; Richter syndrome, 1) with a 48-month PFS of 77% (95% CI 70.2–85.0) (Figure 1A).

In the multivariate analysis, age (≥70 vs. <70 years), CIRS (≥8 vs. <8), Binet stage (C vs. A/B), CrCl, ml/min (≥70 vs. <70), LDH (increased vs. normal), IGHV (unmutated vs. mutated), and del 11q (present vs. absent) did not show a significant impact on PFS, while B-symptoms and TP53 disruption emerged as the only independent factors associated with a significantly shorter PFS (Table 2).

Twelve patients died, seven because of an adverse event (AE; heart failure, 1; severe infection, 5; liver failure, 1), four due to a second malignancy, and one due to disease progression. The 48-month OS rate was 90% (95% CI 84.7–95.3; Figure 1B). High LDH levels (*p* = 0.03), B symptoms (*p* = 0.03), and *TP53* disruption (*p* = 0.04) showed a significant impact on OS in the univariate analysis (Supplementary Table S1). However, none of these factors maintained significance in the multivariate analysis.

### 3.3. Adverse Events

The type and severity of the AEs recorded in this study are described in Supplementary Table S2. Grade 3–4 granulocytopenia, recorded in 27% of patients, was the most common AE leading to dose reduction or transient treatment interruption. At the last follow-up, the daily dose of ibrutinib received by the 80 patients still on treatment was 420 mg in 62 (77.5%), 280 mg in 14 (17.5%), and 140 mg in 5 (5%). Grade ≥3 infections were diagnosed in 18% of patients, and included lower respiratory tract infections in 8%, with three cases of lethal SARS-CoV-2 pneumonia. Any grade cardiovascular AEs were recorded in 30% of patients and included atrial fibrillation in 16% (grade 3–4 atrial fibrillation, 6%). New-onset hypertension was experienced by 13% of patients. Any grade bleeding disorders were observed in 23% of patients. However, severe bleeding events were uncommon (5%) and included cerebral hemorrhage in three cases. Other AEs frequently reported were any grade myalgias and arthralgias (16%), diarrhea (14%), and skin rash (10%). A non-skin second malignancy was diagnosed in 13 (9%) patients (gastric, 3; lung, 2; bladder, 2; breast, 1; mesothelioma, 1; neuroendocrine, 1; thyroid, 1; bowel, 1; hepatic, 1). No new safety signals or unknown/unwitnessed deaths were recorded.

### 3.4. Adverse Events Leading to Permanent Treatment Discontinuation

The main reason leading to the permanent discontinuation of treatment was represented by an AE, recorded in 44 (30.1%) patients. Treatment discontinuations rates due to AEs were 17.8% at 12 months, 23.3% at 24 months, 26.0% at 36 months, and 29.1% at 48 months. The median age of patients who discontinued ibrutinib permanently due to an AE was 78 years (range 56.8–90.2).

**Figure 2:**
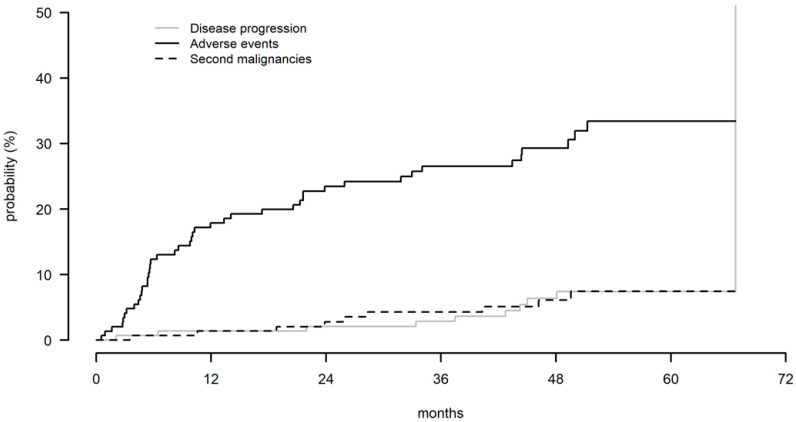
Cumulative rates of treatment discontinuation according to the reasons for treatment discontinuations, disease progression, adverse events, and second malignancies.

Cardiovascular disorders were a common AE, leading to treatment discontinuation (11% of the cases, including atrial fibrillation in 8%), followed by infections (8%), non-skin cancers (6%), and cerebral hemorrhage, 3% (Supplementary Table S2). In the multivariate analysis, the male gender was significantly and independently associated with a higher rate of treatment discontinuations due to AEs (HR: 0.46; *p* = 0.05; Supplementary Table S3). Two other factors showed a significant and independent impact on discontinuations caused by AEs, aged older than 70 years (HR: 5.43; *p* = 0.002), and treatment managed at centers that enrolled less than five patients (HR: 0.51, *p* = 0.04). Based on an age older than 70 years and less than five patients enrolled by the referral centers, we identified three groups of patients. In the low-risk group, which included patients with none of the above risk factors, the rate of discontinuations was 11.8%; in the intermediate-risk group that included patients with one of the two risk factors, the rate was 28.3%, while for patients of the high-risk group who showed both risk factors, the rate of discontinuations was 52.5% (*p* = 0.001; Figure 2).

### 3.5. Prognostic Impact of Treatment Discontinuation

**Figure 3:**
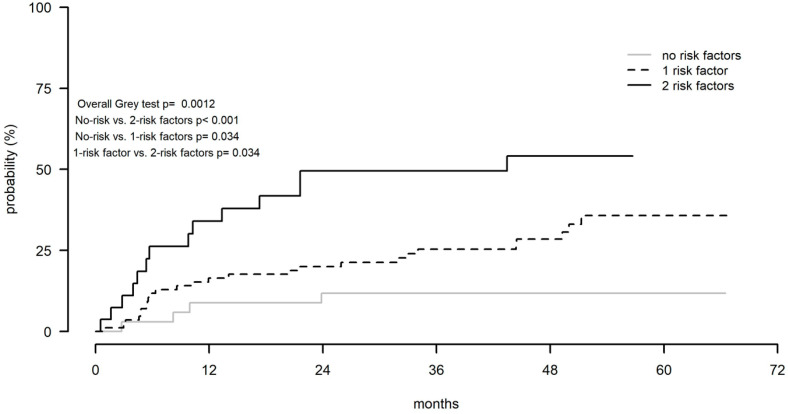
Cumulative rates of treatment discontinuations due to adverse events according to the number of risk factors.

At the time of the last follow-up, 80 (55%) patients, including 31 patients in CR (31/80, 39%; 31/146, 21.2%), were still on ibrutinib, while 66 (45%) discontinued treatment (Supplementary Figure S1). The 48-month cumulative rates of treatment discontinuation due to disease progression, AEs, and second malignancies were 5.6%, 29.1%, and 6%, respectively (Table 3 and Figure 3).

**Figure 4:**
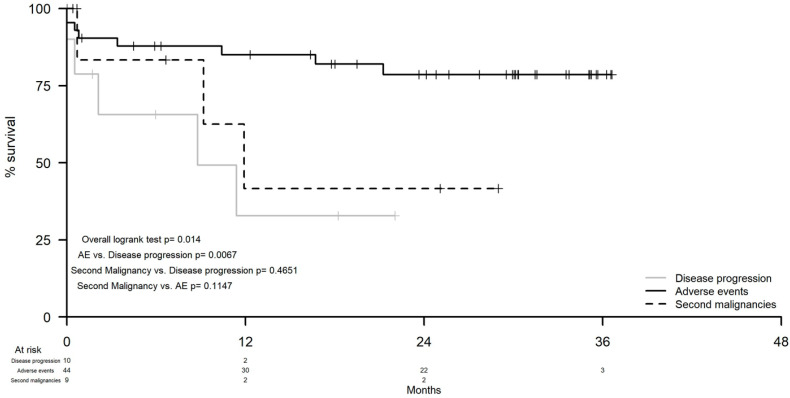
Survival probability from treatment discontinuation according to the reason for discontinuations, disease progression, adverse event, and second malignancy.

The 12-month survival rates of patients who permanently discontinued treatment due to AE, second malignancy, and disease progression were 85% (95% CI: 74.6–96.9), 41.7% (95% CI: 14.7–100.0), and 33% (95% CI: 11.0–98.1), respectively (*p* = 0.01; Figure 4).

The 24-month next treatment-free survival rate of patients who discontinued ibrutinib due to AE was 63% (Supplementary Figure S2).

## 4. Discussion

We investigated the benefit and safety of a front-line treatment with the ibrutinib and rituximab combination in an unfit cohort of CLL patients, defined by a CIRS comorbidity score >6 and/or a reduced renal function. The results of this study confirm the efficacy of this schedule in 146 patients with CLL and with a median age of 73 years. Furthermore, 87% of patients achieved a response, which included a CR in 22.6% of the cases. Moreover, 6.2% of all patients and 27% of CR patients showed a response with uMRD by flow-cytometry. Although the absence of a control arm limits the results of this study, the relatively high CRs and PFS rates we observed, 77% at 48 months, were consistent with those of other trials investigating the efficacy of ibrutinib-based treatments in the front-line setting [4,5,6,7,15,16,17,18,19,20,21,22,23]. The modulation of molecules interacting with the microenvironment produced by the treatment may have favored the fast mobilization of CLL cells [24]. The low rate of disease progressions observed in our study, 10%, further confirmed that the emergence of ibrutinib-resistant subclones is rare in the front-line setting [4,5,6,7].

In this study, PFS was not significantly influenced by IGHV mutational status. Moreover, del (11q) or the achievement of CR did not exert the same beneficial impact on PFS described in other studies [25,26]. As observed in the Alliance trial [6], patients with *TP*53 showed an inferior PFS. That being said, the 48-month PFS of 65% was higher than observed in the past with chemoimmunotherapy in this subset of patients, and is in line with that of other studies with ibrutinib in patients carrying *TP*53 disruption [27,28]. A significantly lower PFS was associated with the presence of B symptoms. This finding underlines the unfavorable impact of symptomatic disease. In the study by Woyach et al., patients treated with ibrutinib and rituximab showed a PFS similar to those who received single-agent ibrutinib [6]. The PFS value we observed with ibrutinib and rituximab was not superior to that described in other studies with an ibrutinib single agent [4,23]. This observation further questions the benefit of adding rituximab to ibrutinib.

In this patient population, treatment discontinuations due to AE were frequent, with a 48-month cumulative rate of 29.1%. This was not an unexpected finding in patients already older and unfit at baseline. In two trials that included younger patients, the discontinuation rates due to AEs were 19.1% at 4 years and 21% at 5 years, respectively [8,22]. In a retrospective analysis that included 616 patients with CLL, toxicities were also the most common reason for treatment discontinuation [10]. Variable rates of ibrutinib discontinuations have also been reported in real-world studies [29,30,31,32,33]. It is noteworthy that treatment discontinuations rates due to AEs were lower with fixed-duration venetoclax combined with obinutuzumab or rituximab [34,35].

An intriguing finding was the relatively high 12-month survival rate, 85%, and the 24-month next treatment-free survival, 63%, of patients who discontinued treatment due to AE. Similar favorable outcomes have also been described in other studies [7,9,10].

Atrial fibrillation is a well-known AE associated with the use of ibrutinib [30]. The rate of any grade atrial fibrillation was 16%, similar to that of other studies that included older patients treated front-line with ibrutinib [4,6].

Atrial fibrillation was the reason for treatment discontinuation in 8% of patients, a higher rate than previously reported [5,6,22,36]. The characteristics of our patient population may have influenced the discontinuations rates due to atrial fibrillation and also to infections, in 8%. The impact of ibrutinib on cellular immunity has been extensively investigated, with conflicting results. While pre-clinical data described multiple inhibitory effects of ibrutinib on the activity of natural killer cells and macrophages [37,38], recent data suggest that ibrutinib may induce an in vivo immune modulation, with a TH2/TH1 shift in the peripheral blood lymphocytes that is more pronounced in IGHV unmutated and CR patients [39].

A higher rate of discontinuations due to AEs was observed among male patients. To the best of our knowledge, a relationship between sex and treatment discontinuation due to AEs has not been reported in patients treated with ibrutinib. The higher incidence of atrial fibrillation described in males may have had an impact on the increased rate of discontinuations. Older age, over 70 years, and treatment managed at centers that enrolled less than five patients were also associated with an increased rate of discontinuations due to AEs. The presence of both risk factors was associated with a 52.5% discontinuation rate. Older age plays an important role in developing AEs leading to treatment discontinuation. Increasing age is a risk factor for cardiovascular disorders, and the incidence of most cancers also increases with age. Moreover, functions of the immune system decline with age predisposing infections. As previously suggested [40,41], close collaboration withardio-oncologists and infectious disease specialists should be considered in order to avoid treatment discontinuations due to the toxicities of the targeted agents.

Long-term follow-up data from studies will allow for evaluating whether second-generation BTK inhibitors or a time-limited therapy with venetoclax could be preferable in unfit and older patients.

## 5. Conclusions

In conclusion, this study shows that the ibrutinib and rituximab combination is an effective front-line treatment with sustained disease control in more than half of unfit and elderly patients with CLL. However, our data highlights the high rate of treatment discontinuations due to AEs and suggests careful monitoring to prevent and manage AEs in this patient population.

## Figures and Tables

**Table 1:** Baseline clinical and biologic characteristics of patients.

Heading		N (%)
No of patients		146
Gender	male	88 (60.3)
	female	58 (39.7)
Age median (range)		73 (37–88)
Age	<70 yrs	47 (32.2)
	≥70 yrs	99 (67.8)
B symptoms	present	26 (18.7)
	absent	113 (81.3)
Binet Stage	A–B	92 (63.0)
	C	54 (37.0)
B2M	normal	27 (25.2)
	increased	80 (74.8)
LDH	normal	103 (71.0)
	increased	42 (29.0)
CIRS	median (range)	6 (1–21)
	≥8	6 (8)
CrCl, mL/min	median (range)	62.7 (0.80–152.00)
	<70 mL/min	87 (64.0)
ECOG PS	0	90 (62.1)
	1–2	55 (37.9)
Patients with IgG levels	>400 mg/dL	24 (17.5)
	≤400	113 (82.5)
FISH aberrations		
	del13q	42 (29.1)
	del 11q	21 (14.6)
	trisomy 12	24 (16.7)
	del 17q	13 (9.0)
	no aberrations	44 (30.6)
*TP*53 disruption ^a^	present	32 (22.2)
	absent	112 (77.8)
IGHV	unmutated	83 (56.9)
	mutated	63 (43.1)

B2M—β2-Mmcroglobulin; LDH—lactate dehydrogenase; CIRS—Cumulative Illness Rating Scale score; CrCl—creatinine clearance; ECOG PS—Eastern Cooperative Oncology Group performance-status; FISH—fluorescence in situ hybridization; IGHV—immunoglobulin heavy-chain variable region gene; ^a^ Del17p and/or *TP*53 mutation.

**Table 2:** Impact of baseline factors on progression-free survival: univariate and multivariate analysis.

Variables	Univariate Analysis		Multivariate Analysis	
	Hazard Ratio (95% CI)	*p*-Value	Hazard Ratio (95% CI)	*p*-Value
Age ≥70 vs. <70 years	2.09 (0.91–4.82)	0.08	1.34 (0.51–3.54)	0.54
CIRS: ≥8 vs. <8	1.34 (0.54–3.31)	0.52	-	-
Binet stage: C vs. A/B	1.01 (0.50–2.07)	0.96	1.48 (0.63–3.52)	0.37
B symptoms: present vs. absent	2.37 (1.10–5.11)	0.02	2.91 (1.18–7.17)	0.02
CrCl, mL/min ≥70 vs. <70	0.47 (0.19–1.17)	0.10	0.52 (0.19–1.49)	0.23
LDH increased vs. normal	1.46 (0.70–3.04)	0.30	-	-
IGHV unmutated vs. mutated	1.75 (0.83–3.71)	0.13	1.54 (0.63–3.78)	0.34
Del 11q present vs. absent	2.34 (1.03–5.32)	0.04	2.13 (0.76–5.98)	0.15
*TP*53 disruption present vs. absent	1.74 (0.82–3.73)	0.15	2.47 (1.07–5.74)	0.03

CIRS—Cumulative Illness Rating Scale; CrCl—creatinine clearance; IGHV—immunoglobulin heavy-chain variable region gene.

**Table 3:** Reasons for treatment discontinuation.

Reason for Treatment Discontinuation	No. Patients	% Patients	48-Months Cumulative Incidence (95% CI)	Median Age, Years (Range)
Disease progression	10	6.8%	5.6% (1.5–9.6)	76 (57–85)
Adverse events	44	30.1%	29.1% (21.5–36.6)	78 (57–90)
Second malignancies	9	6.2%	6.0% (1.9–10.1)	76 (56–81)

## Data Availability

Study data were collected and managed using REDCap (Research Electronic Data Capture; Supplementary Material) a web-based software platform designed to support data capture for research studies. The data presented in this study are available on request. Qualified researchers may request access to anonymized patient data and study documents. Details on sharing criteria and processes for requesting access to data can be required to a.piciocchi@gimema.it.

## Supplementary Materials

The following are available online at www.mdpi.com/2072-6694/14/1/207/s1. Figure S1: Patients dispositions. Figure S2: Next treatment-free survival for patients who discontinued ibrutinib due to an adverse event. Table S1: Impact of baseline factors on overall survival: univariate analysis. Table S2: Adverse events. Table S3: Impact of clinical and biologic characteristics of patients on treatment discontinuation due to adverse events: univariate and multivariate analysis.

## Abbreviations

CLL	Chronic Lymphocytic Leukemia
OR	Overal response rate
CR	Complete Response
CRi	Complete Response with incomplete bone marrow recovery
PR	Partial response
PR-L	Partial response with Lymphocytosis
MRD	Minimal residual disease
UMRD	Undetectable minimal residual disease
PFS	Progression free survival
OS	Overall survival
IGHV	ImmunoGlobulin heavy-chain variable region gene;
AE	Adverse event
GIMEMA	Gruppo Italiano Malattie EMatologiche dell’Adulto
B2M	β2-Mmcroglobulin
LDH	Lactate DeHydrogenase
CIRS	Cumulative Illness Rating Scale score
CrCl	Creatinine clearance
ECOG PS	Eastern Cooperative Oncology Group performance-status
FISH	Fluorescence in situ hybridization
TP53	Tumor protein p53
ITT	Intention to treat
EOCT	End of combination therapy
Ig	Immunoglobulins
