# Supplementary material for: Efficacy of Front-Line Ibrutinib and Rituximab Combination and the Impact of Treatment Discontinuation in Unfit Patients with Chronic Lymphocytic Leukemia: Results of the Gimema LLC1114 Study
Source: Cancers (Basel). 2021 Dec 31;14(1):207. doi: 10.3390/cancers14010207 (PMC8750939; doi:10.3390/cancers14010207)
Supplement: Supplementary file 1 [file cancers-14-00207-s001.zip › cancers-1526427-supplementary.pdf]

SUPPLEMENTARY MATERIAL

Statistical analysis

Study data have been collected and managed using REDCap electronic data capture tools (1, 2). (Supplementary Material) hosted at Fondazione GIMEMA Franco Mandelli ONLUS. REDCap (Research Electronic Data Capture) is a secure, web-based software platform designed to support data capture for research studies, providing 1) an intuitive interface for validated data capture; 2) audit trails for tracking data manipulation and export procedures; 3) automated export procedures for seamless data downloads to standard statistical packages, and 4) procedures for data integration and interoperability with external sources.

1. Obeid JS, McGraw CA, Minor BL, Conde JG, Pawluk R, Lin M, Wang J, Banks SR, Hemphill SA, Taylor R, Harris PA. Procurement of shared data instruments for Research Electronic Data Capture (REDCap). J Biomed Inform. 2013;46(2):259-65. doi: 10.1016/j.jbi.2012.10.006.

2. Harris PA, Taylor R, Minor BL, Elliott V, Fernandez M, O'Neal L, McLeod L, Delacqua G, Delacqua F, Kirby J, Duda SN; REDCap Consortium. The REDCap consortium: Building an international community of software platform partners. J Biomed Inform. 2019l;95:103208. doi: 10.1016/j.jbi.2019.103208.

.

Table S1.
Impact of baseline factors on overall survival: univariate analysis.

Variables	Univariate analysis	
	**HR (95% CI)**	**p**
**Age ≥70 vs <70 years**	2.44 (0.69-8.58)	0.16
**CIRS ≥8 vs <8**	0.84 (0.19-3.74)	0.81
**Binet stage: C vs. A/B**	1.47 (0.55-3.97)	0.44
**B symptoms present vs absent**	3.12 (1.11-8.81)	0.03
**CrCl, ml/min ≥70 vs <70**	0.42 (0.12-1.50)	0.18
**LDH increased vs normal**	2.97 (1.11-7.95)	0.03
**IGHV unmutated vs mutated**	1.28 (0.46-3.52)	0.63
**Del 11q present vs. absent**	1.62 (0.45-5.91)	0.46
**Del17p and/or *TP*53 mutation present vs. absent**	2.89 (1.00-8.33)	0.04

Abbreviations: CIRS, Cumulative Illness Rating Scale score; CrCl, creatinine clearance; HR, Hazard Ratio; IGHV; immunoglobulin heavy-chain variable region gene

Table S2. Adverse events.

Adverse events (AEs)	All grade N (%)	Grade 1-2 N (%)	Grade 3-4 N (%)	Grade 5 N (%)	AEs leading to discontinuation N (%)
**HEMATOLOGIC**	**59 (40)**	**13 (9)**	**46 (32)**	**0 (0)**	**5 (3)**
Anemia	6 (4)	4 (3)	2 (1)	0 (0)	1 (1)
Neutropenia	40 (27)	1 (1)	39 (27)	0 (0)	3 (2)
Thrombocytopenia	12 (8)	8 (5)	4 (3)	0 (0)	1 (1)
Neutrophilia	1 (1)	0 (0)	1 (1)	0 (0)	0 (0)
**CARDIOVASCULAR**	**44 (30)**	**24 (16)**	**19 (13)**	**1 (1)**	**16 (11)**
Atrial fibrillation	23 (16)	14 (10)	9 (6)	0 (0)	11 (8)
Tachycardia	5 (3)	5 (3)	0 (0)	0 (0)	0 (0)
Bradycardia	2 (1)	1 (1)	1 (1)	0 (0)	0 (0)
Cardiac failure	3 (2)	0 (0)	2 (1)	1 (1)	2 (1)
Ischemic disorders	5 (3)	1 (1)	4 (3)	0 (0)	1 (1)
Other cardiologic events (1)	6 (4)	3 (2)	3 (2)	0 (0)	2 (1)
**GASTROENTERIC**	**44 (30)**	**38 (26)**	**6 (4)**	**0 (0)**	**2 (1)**
Abdominal pain	5 (3)	5 (3)	0 (0)	0 (0)	0 0()
Diarrhea	21 (14)	18 (12)	3 (2)	0 (0)	0 (0)
Nausea/vomiting	7 (5)	7 (5)	0 (0)	0 (0)	1 (1)
Gastritis	3 (2)	2 (1)	1 (1)	0 (0)	0 (0)
Other gastroenteric disorder (2)	8 (5)	6 (4)	2 (1)	0 (0)	1 (1)
**RENAL(5)**	**4 (3)**	**3 (2)**	**1 (1)**	**0 (0)**	**0 (0)**
**BLEEDING DISORDERS**	**33 (23)**	**26 (18)**	**7 (5)**	**0 (0)**	**5 (3)**
Cerebral bleeding	5 (3)	0 (0)	5 (3)	0 (0)	5 (3)
Contusion, ecchymosis mucosal bleeding, hematoma.	28 (19)	26 (18)	2 (1)	0 (0)	0 (0)
**CUTANEOUS-ADNEXAL TOXICITIES**	**29 (20)**	**26 (18)**	**3 (2)**	**0 (0)**	**1 (1)**
Rash	14 (10)	11 (8)	3 (2)	0 (0)	1 (1)
Skin and nail lesions	3 (2)	3 (2)	0 (0)	0 (0)	0 (0)
Other skin disorders	12 (8)	12 (8)	0 (0)	0 (0)	0 (0)
**OCULAR TOXICITY (5)**	**6 (4)**	**5 (3)**	**1 (1)**	**0 (0)**	**0 (0)**
**INFUSIONE RELATED REACTIONS**	**15 (10)**	**13 (9)**	**2 (1)**	**0 (0)**	**1 (1)**
**FATIGUE**	**11 (8)**	**8 (5)**	**3 (2)**	**0 (0)**	**1 (1)**
**HEADACHE**	**8 (5)**	**7 (5)**	**1 (1)**	**0 (0)**	**0 (0)**
**FALLS WITH FRACTURE**	**13 (9)**	**8 (5)**	**5 (3)**	**0 (0)**	**1 (1)**
**OEDEMA**	**8 (5)**	**7 (5)**	**1 (1)**	**0 (0)**	**0 (0)**
**INFECTIONS**	**105 (72)**	**74 (51)**	**26 (18)**	**5 (3)**	**12 (8)**
Upper respiratory tract	9 (6)	9 (6)	0 (0)	0 (0)	0 (0)
Lower respiratory tract.(5)	30 (21)	16 (11)	12 (8)	2 (1)	7 (5)
Urogenital	15 (10)	11 (8)	4 (3)	0 (0)	0 (0)
Mucocutaneous and adnexal	20 (14)	16 (11)	4 (3)	0 (0)	0 (0)
Gastroenteric	4 (3)	3 (2)	1 (1)	0 (0)	0 (0)
CNS.(6)	4 (3)	0 (0)	2 (1)	2 (1)	4 (3)
Sepsis	3 (2)	0 (0)	2 (1)	1 (1)	1 (1)
Viral.(7)	20(14)	19 (13)	1 (1)	0 (0)	0 (0)
**PYREXIA**	**19 (13)**	**18 (12)**	**1 (1)**	**0 (0)**	**0 (0)**
**PAIN**	**37 (25)**	**34 (23)**	**3 (2)**	**0 (0)**	**0 (0)**
Muscle spasm	7 (5)	7 (5)	0 (0)	0 (0)	0 (0)
Arthralgias and myalgias	24 (16)	21 (14)	3 (2)	0 (0)	0 (0)
Pain, site not specified	6 (4)	6 (4)	0 (0)	0 (0)	0 (0)
**PSYCHIATRIC**	**10 (7)**	**7 (5)**	**3 (2)**	**0 (0)**	**0 (0)**
Depression	4 (3)	2 (1)	2 (1)	0 (0)	0 (0)
Other symptoms(8)	6 (4)	6 (0)	0 (0)	0 (0)	0 (0)
**NEUROLOGICAL DISORDERS (9)**	**7 (5)**	**5 (3)**	**2 (1)**	**0 (0)**	**0 (0)**

**METABOLIC**[10]	**13 (9)**	**5 (3)**	**7 (5)**	**1 (1)**	**0 (0)**
**CANCERS**	**17 (12)**	**4 (3)**	**9 (6)**	**4 (3)**	**9 (6)**
Non-skin cancers	13 (9)	2 (1)	7 (5)	4 (3)	9 (6)
Non melanoma skin cancers	4 (3)	2 (1)	2 (1)	0 (0)	0 (0)
**THROMBOEMBOLISM**	**5 (3)**	**4 (3)**	**1 (1)**	**0 (0)**	**0 (0)**

[1]Other cardiac events: Hypotension, 4 patients; pericarditis, 2.[2]Other gastro enteric disorders: constipation, 2; dysphagia, 1; dyspepsia, 2; intestinal pneumatosis, 1; not specified, 1.[3]Renal disorders: Neurogenic bladder, 1; renal failure, 1; urinary incontinence, 1.[4] Ocular toxicity: Vision blurred, 1; eye burns, 2; not specified, 1; conjunctivitis, 1; cataract, 1.[5]Lower respiratory tract infection (LRTI), pneumonia, 20 cases; pneumonia leading to treatment discontinuation, 7 cases (Covid-19, 3; aspergillus, 1; cryptosporidium, 1, tuberculosis 1; not specified agent, 1); acute bronchitis, 6.[6] CNS infections: encephalitis, 3; brain abscess, 1.[7] Viral infections: influenza, 8, herpes simplex, 2; herpes varicella zoster, 4; cytomegalovirus, 1; unknown viral infection, 2, not specified, 3. [8] Anxiety, 1;insomnia, 2: tinnitus, 2;unspecified, 1. [9] Neurological disorders: Peripheral sensorimotor neuropaty,1; neuralgia, 2; gaiter disturbance;1; aphasia, 1; neuritis, 1; demyelinating polyneuropathy, 1. [10] Metabolic: transaminases increased, 4; liver failure, 1; amylases, increased, 1; hyperglycemia, 2; hypercalcemia, 2; hyponatremia, 1; hyperuricemia, 1; iron deficiency, 1.

Table S3. Impact of clinical and biologic characteristics of patients on treatment discontinuation due to adverse events: univariate and multivariate analysis

Variables	Univariate analysis		Multivariate analysis	
	**HR (95% CI)**	**p**	**HR (95% CI)**	**p**
**Age**<br>**≥70 vs <70 years**	4.46 (1.75-11.36)	0.002	5.43 (1.89-15.6)	0.002
**Gender**<br>**F vs M**	0.47 (0.24-0.92)	0.03	0.46 (0.21-1.01)	0.05
**CIRS**<br>**>8 vs ≤8**	1.81 (0.88-3.71)	0.11	1.93 (0.60-6.23)	0.27
**Binet**<br>**C vs A/B**	1.63 (0.91-2.95)	0.10	1.08 (0.49-2.38)	0.84
**B symptoms**<br>**present vs absent**	0.55 (0.22-1.41)	0.21	0.41 (0.13-1.31)	0.13
**LDH**<br>**increased vs normal**	0.89 (0.45-1.79)	0.76	1.03 (0.49-2.19)	0.94
**IGHV**<br>**unmutated vs mutated**	0.74 (0.41-1.32)	0.30	0.75 (0.38-1.50)	0.42
**Del17p and/or *TP*53 mutation**<br>**present vs absent**	1.13 (0.55-2.30)	0.74	1.09 (0.49-2.45)	0.84
**Patients from centers that enrolled**<br>**≥5 vs <5 patients**	0.65 (0.35-1.21)	0.18	0.51 (0.26-0.99)	0.04

Abbreviations: CIRS, Cumulative Illness Rating Scale score; HR: Hazard Ratio; IGHV; immunoglobulin heavy-chain variable region gene

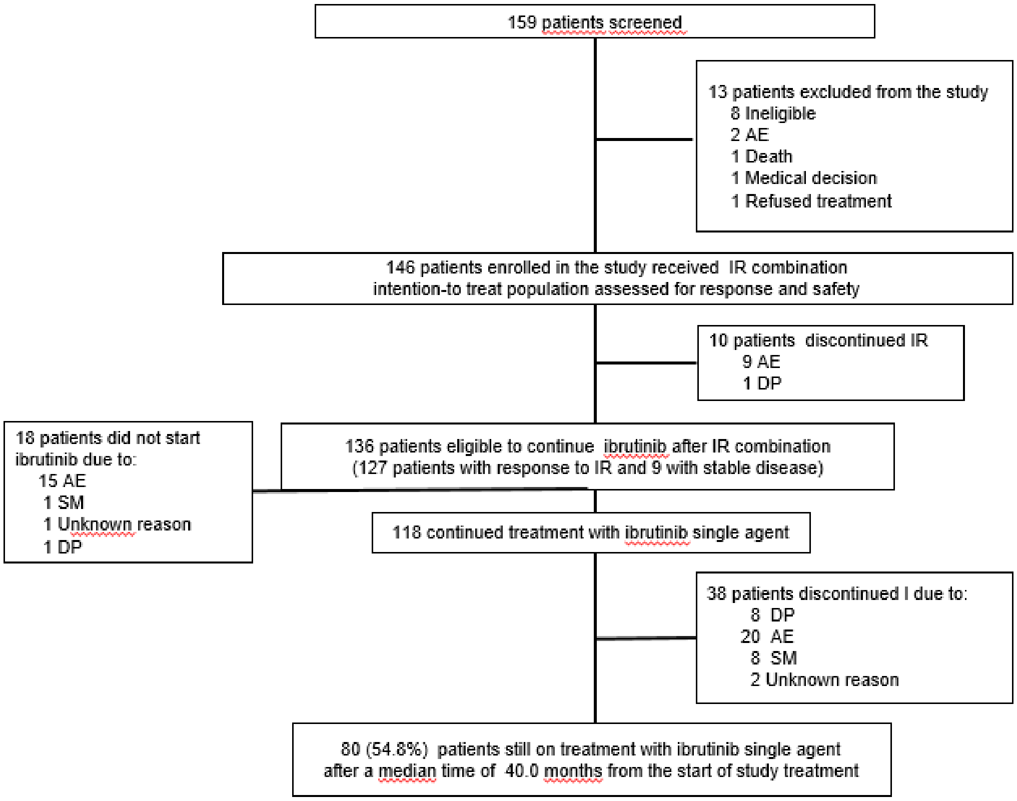

Supplementary Fig. 1. Patients disposition

Abbreviations: AE, adverse event; SM, second malignancy; DP, disease progression; IR, ibrutinib and rituximab.

Figure S2.

Next treatment-free survival for patients who discontinued ibrutinib due to an adverse event.

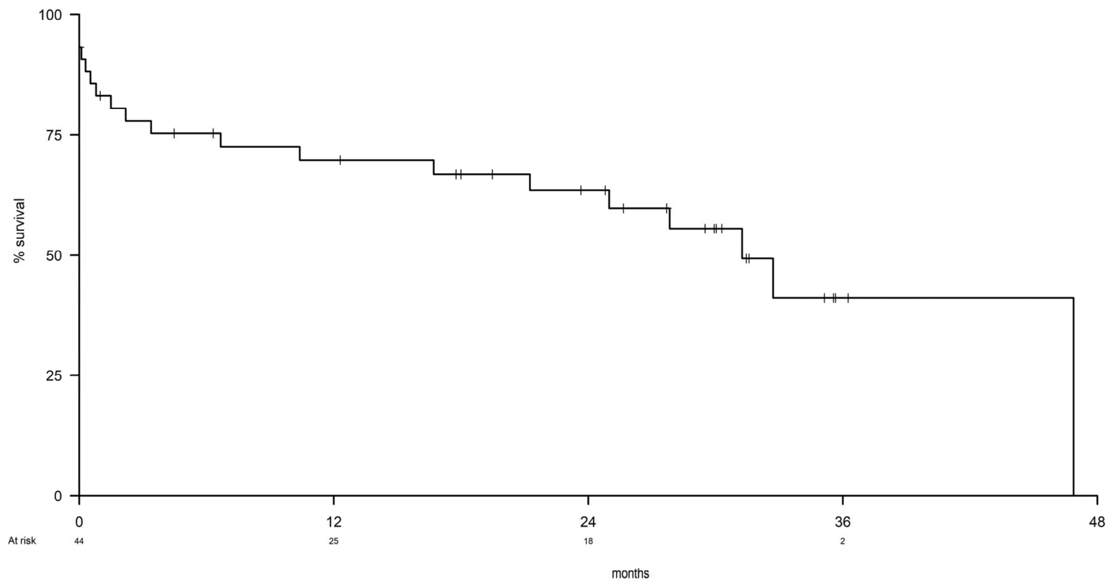

LIST FOR THE ABBREVIATIONS

CLL	Chronic Lymphocytic Leukemia
OR	Overal response rate
CR	Complete Response
CRi	Complete Response with incomplete bone marrow recovery
PR	Partial Response
PR-L	Partial Response with Lymphocytosis
MRD	Minimal Residual Disease
UMRD	Undetectable Minimal Residual Disease
PFS	Progression Free Survival
OS	Overall Survival
IGHV	ImmunoGlobulin Heavy-chain Variable region gene;
AE	Adverse event
GIMEMA	Gruppo Italiano Malattie EMatologiche dell'Adulto

B2M	β2-Mmcroglobulin
LDH	Lactate DeHydrogenase
CIRS,	Cumulative Illness Rating Scale score
CrCl,	Creatinine Clearance
ECOG PS	Eastern Cooperative Oncology Group performance-status
FISH	Fluorescence in situ hybridization
TP53	Tumor Protein p53
ITT	Intention to treat
EOCT	End of Combination therapy
Ig	Immunoglobulins
